# Reframing obstetric care through a trauma‐informed lens: A narrative review of trauma‐informed principles and clinical applications

**DOI:** 10.1002/pmf2.70081

**Published:** 2025-08-05

**Authors:** Brooke E. Schroeder, Jeffrey A. Kuller, Sarah K. Dotters‐Katz

**Affiliations:** ^1^ Duke University School of Medicine, Duke University Durham North Carolina USA; ^2^ Department of Obstetrics and Gynecology Duke University Medical Center Durham North Carolina USA

**Keywords:** patient‐centered care, perinatal care, prenatal care, trauma‐informed care

## Abstract

**Importance:**

Trauma is exceedingly common in the United States adult population; therefore, obstetric patients have more than likely experienced trauma within their lifetime and are at risk of re‐traumatization in the perinatal period. Trauma‐informed care is a model that seeks to address patient trauma histories and how that affects their ability to seek healthcare, as well as try to mitigate re‐traumatization through medical care. However, there is little dedicated curricula around this topic, and many providers feel unequipped to implement trauma‐informed care. Therefore, the goal of this narrative review is to provide an overview of trauma‐informed care in obstetrics and highlight actionable steps that providers can take to integrate trauma‐informed care in their practice.

**Methods:**

We conducted a literature search (2005–2025) on PubMed and OVID using the terms (“trauma‐informed care” OR “trauma‐based care” OR “trauma‐sensitive care”) AND (“pregnancy” OR “pregnant women” OR “prenatal care” OR “perinatal care” OR “labor and delivery” OR “childbirth” OR “birth” OR “obstetrics”). A total of 482 PubMed and 167 OVID articles were screened for relevance. Additionally, key society guidelines and existing trauma‐informed care curricula were reviewed to provide context and recommendations.

**Results:**

Our findings indicate that traumatic life events (TLEs) are highly prevalent among pregnant individuals, particularly among adolescents, racial and sexual minorities, and those of lower socioeconomic status. Pregnancy increases risk for intimate partner violence and mood disorders, and one‐third of women report birth itself as traumatic, with higher rates of distress after emergency cesarean delivery (up to 80.6%). A history of trauma is associated with adverse perinatal outcomes including heightened risk of preterm birth, pregnancy loss, postpartum depression, and post‐traumatic stress disorder. Trauma also affects maternal‐child bonding, breastfeeding success, and long‐term child development. Evidence supports that trauma‐informed care can mitigate these outcomes. Strategies including patient‐centered communication, doula support, group prenatal care, and multidisciplinary perinatal mental health programs have shown improved engagement in care, reduced cesarean risk, and better psychological outcomes for pregnant patients.

**Conclusions:**

Trauma and traumatic stress are highly prevalent in the obstetric population, with unmanaged stress contributing to worsened maternal and fetal outcomes. Implementing both individual and institutional interventions can enhance patient‐centered care and support resilience among trauma survivors. By mitigating adverse health effects, trauma‐informed care serves as a vital strategy for advancing health equity and reducing disparities in maternal morbidity and mortality.

## 1 INTRODUCTION

### 1.1 What is trauma‐informed care?

Trauma is commonly defined as the experience of or exposure to an event involving actual or threatened death, serious injury, or sexual violence [1]. However, trauma is a broad and pervasive experience, also encompassing emotional abuse, neglect, loss, and systemic abuses of power. Trauma‐informed care (TIC) is a model that encourages providers to realize the widespread impact of trauma, recognize the signs and symptoms of traumatic stress, respond with thoughtfulness and empathy, and actively resist re‐traumatization of patients [2]. This framework teaches providers to interpret a patient's behavior within the context of their past experiences rather than making assumptions or judgments. In obstetrics and gynecology (OB/GYN), TIC is especially relevant, as much of this care occurs in intimate and vulnerable settings where physical exposure and examinations may trigger traumatic memories, or emergent scenarios may impair a patient's sense of autonomy.

### 1.2 Trauma and its influence on health outcomes

Traumatic life events (TLEs) activate the hypothalamic‐pituitary‐adrenal axis, triggering the release of stress hormones such as cortisol and catecholamines [3]. These hormones are essential for helping the body respond effectively in moments of acute crisis. However, chronically elevated stress hormone levels—such as in situations of ongoing abuse, unstable living environments, or systemic discrimination—can lead to long‐term physiological consequences [4, 5]. Over time, this chronic activation can cause dysregulation of the body's stress response systems and increased inflammation [6]. Beyond physical health, disruptions in stress regulation can also affect emotional regulation, impair communication, and complicate interpersonal relationships [7, 8].

Adverse childhood experiences (ACEs)—which include abuse, neglect, and household dysfunction before age 18—are among the most well‐studied examples of trauma leading to negative long‐term consequences. These experiences contribute to negative health effects in a dose‐dependent manner: the more ACEs a person endures, the greater their risk for psychiatric illness, chronic diseases (e.g., diabetes, cardiovascular disease), and harmful behaviors such as substance use [9, 10, 11].

Importantly, TLEs are not limited to childhood. Traumatic experiences in adulthood—such as intimate partner violence (IPV), sexual assault, combat exposure, or medical trauma—can independently disrupt neuroendocrine regulation and lead to poor health outcomes [12]. These effects can compound as many people who experience trauma in early life are susceptible to re‐traumatization, increasing the risk of health complications [13]. Therefore, it is imperative to address traumatic stress and its downstream effects in order to provide effective, holistic patient care.

### 1.3 Trauma‐informed care and health disparities

Both trauma and racial identity are independently linked to adverse medical outcomes, and TIC offers a promising approach to reducing racial health disparities—particularly the pervasive and striking disparities in maternal and neonatal morbidity and mortality [14, 15].

People of racial or ethnic minorities are significantly more likely to have experienced TLEs [16, 17, 18]. These experiences are often intensified by ongoing racial discrimination and culturally insensitive care, which can heighten trauma‐related symptoms and contribute to both psychological and physiological harm [19, 20]. In addition to ongoing stigmatization, racial trauma encompasses historical and intergenerational trauma rooted in systemic racism—such as residential segregation, economic marginalization, and longstanding policies of social exclusion [21]. These structural forces shape the social determinants of health that contribute to health disparities and expose marginalized communities to external stressors such as violence and poverty.

Amid the ongoing maternal mortality crisis in the United States, TIC represents a critical pathway toward health equity in perinatal care. By acknowledging and addressing the effects of chronic and cumulative trauma, TIC can be implemented at both individual and institutional levels to promote safer, more respectful, and culturally responsive care. This approach has the potential not only to improve clinical interactions and patient trust but also to reduce adverse outcomes for marginalized populations by reforming systems that have historically perpetuated inequity.

### 1.4 Trauma in obstetrics

Pregnancy and childbirth can be uniquely traumatizing, as it challenges a person's sense of control and bodily autonomy, particularly in medical settings. These experiences, when layered with historical or current traumatic stressors, may heighten a patient's risk of re‐traumatization during perinatal care [22]. Importantly, pregnant individuals who belong to marginalized populations are disproportionately affected by trauma and face unique challenges within perinatal care settings, such as mistreatment by healthcare providers [23]. Moreover, pregnancy can involve inherently traumatic events, such as pregnancy loss, stillbirth, or distressing birth experiences—each of which can leave lasting psychological and physical effects. Additionally, these events can have profound implications for how patients experience and engage with care in subsequent pregnancies.

As the effect of trauma is so pervasive, and survivors may be hesitant to disclose their past to providers, TIC has become a universally recommended approach for every patient encounter [24]. However, there is little formal education on TIC and many obstetric providers do not feel confident in providing trauma‐sensitive care [25, 26, 27]. Barriers to implementation include limited training opportunities, time constraints that limit the ability to provide patient‐centered communication, and a lack of institutional support or resources to adopt trauma‐informed practices consistently. These gaps underscore the urgent need for accessible education and systemic changes to integrate TIC effectively into obstetric care. This review examines the prevalence and effects of trauma in the perinatal period and provides practical guidelines for integrating TIC into obstetric practice.

## 2 METHODS

We conducted a literature search (2005–2025) on PubMed and OVID using the terms (“trauma‐informed care” OR “trauma‐based care” OR “trauma‐sensitive care”) AND (“pregnancy” OR “pregnant women” OR “prenatal care” OR “perinatal care” OR “labor and delivery” OR “childbirth” OR “birth” OR “obstetrics”). A total of 482 PubMed and 167 OVID articles were screened for relevance, leading to the exclusion of 319 PubMed articles and 91 OVID articles.

Additionally, key society guidelines and existing TIC curricula were reviewed to provide context and recommendations. This narrative review was structured in accordance with the SANRA (Scale for the Assessment of Narrative Review Articles) guidelines to ensure clarity, scientific rigor, and relevance (Supporting Information S1) [28].

## 3 RESULTS

### 3.1 Prevalence of trauma in the perinatal period

Prior research has shown that TLEs prior to pregnancy are exceedingly common, with about one‐third of pregnant people reporting at least one TLE (Table 1) [29, 30]. Many patients report multiple TLEs, highlighting how prior traumatic stress can increase vulnerability to future trauma, particularly in perinatal care settings [31]. Sexual trauma is often one of the most commonly reported TLEs, which can contribute to unique vulnerabilities in labor and perinatal care [32].

**TABLE 1:** Sample of traumatic life events included in the Life Stressor Checklist–Revised (LSC‐R) **[** 33]. The LSC‐R is a validated and widely used tool for assessing lifetime exposure to traumatic events. This table presents a subset of representative items included in the questionnaire. Of note, the LSC‐R does not include all possible trauma etiologies, such as childhood exposure to family mental illness or substance use disorders.

Serious disaster (e.g., earthquake, hurricane, large fire, explosion)
Serious car accident
Experience of incarceration or close family member incarcerated
Parental divorce/separation or personal experience of divorce/separation
Serious physical or mental illness
Pregnancy loss
Separation from child against your will (e.g., loss of custody, kidnapping)
Witnessing violence between family members
Physically or sexually assaulted

The prevalence of TLEs is even higher for patients of lower socioeconomic status and those who identify as racial, ethnic, and/or sexual minorities. These communities are disproportionately exposed to structural and interpersonal trauma, including racial discrimination, housing and economic insecurity, and reduced access to healthcare [18, 34]. Residential racial segregation—driven by historic and ongoing policies such as redlining—has concentrated poverty and violence in many communities of color, further compounding the risk of TLEs [35, 36, 37, 38].

Due to these structural inequities, Non‐Hispanic Black patients experience higher rates of lifetime trauma exposure compared to Non‐Hispanic White patients (Roberts: 8.7% vs. 7.4%; Alegría: 7.8% vs. 6.9%) and have an increased risk of developing post‐traumatic stress disorder (PTSD) (aOR = 1.22; 95% CI: 1.05–1.43) [16, 17]. Despite greater exposure to trauma, patients of color are less likely to access or be offered appropriate mental health resources. One study found that although pregnant Black patients reported higher exposure to trauma, they were less likely to seek mental health treatment than their White counterparts (odds ratio [OR] = 0.3; *p* < 0.001) [39]. Additionally, individuals from marginalized groups frequently encounter discrimination within healthcare settings, which itself is a source of trauma. During pregnancy, patients of color report higher levels of racial discrimination compared to Non‐Hispanic White patients [40]. These findings highlight the cumulative burden of racial trauma, which includes interpersonal mistreatment and broader systemic injustices.

Beyond preexisting TLEs, pregnancy itself can increase the risk of ongoing trauma, including IPV and mood disorders. Pregnancy increases the risk of the occurrence as well as the severity of IPV, with the risk even higher for unplanned pregnancies (odds ratio [OR] = 1.66; 95% CI: 1.20–1.31) [41, 42]. Pregnant adolescents are particularly vulnerable, with one study reporting over 80% prevalence of TLEs and 75% of the sample experienced IPV [43]. Homicide is one of the leading causes of death among pregnant individuals, underscoring the extreme consequences of unrecognized or unaddressed IPV [44]. In addition to the risk of physical harm, the increased emotional stresses of pregnancy and preparing for the birth of a child can lead to increased incidence of depression and anxiety [43]. These physical and emotional burdens of TLEs predispose pregnant individuals to future stressors and increase their risk of long‐term effects of trauma.

The healthcare setting is intended to be a place of safety for all patients; however, it can be a significant source of traumatic experiences for pregnant individuals, particularly when it comes to labor and childbirth. Obstetric violence is defined as the mistreatment or disrespect of birthing individuals through verbal abuse, unconsented or inadequately consented procedures, neglect, or inadequate pain management [45, 46]. Prior studies indicate that one‐third of women describe their birth as traumatic, with nearly 1 in 5 also reporting experiences of obstetric violence or mistreatment [47, 48]. Reported rates of birth trauma have been shown to be highest among patients who undergo emergency cesarean delivery (80.6% of patients) or instrumental vaginal delivery (62.2%) [29]. Other studies similarly report elevated rates of traumatic stress symptoms following emergency cesarean birth [29, 49, 50].

Factors affecting patient's experience of birth—including fear for their own or their baby's life, loss of autonomy, and poor communication—can contribute to lasting psychological distress, including symptoms of PTSD [51, 52]. Symptoms of traumatic stress related to childbirth are also even more common in those with a history of PTSD [53]. This increased vulnerability may be due to an exaggerated physiological stress response, as well as difficulties with interpersonal relationships that can complicate communication and trust with healthcare providers, ultimately diminishing their sense of autonomy and safety during the birth experience [54, 55]. While labor and birth are inherently unpredictable and sometimes life‐threatening, trauma‐informed approaches that prioritize patient autonomy and provide clear, compassionate communication can help reduce fear and mitigate the effect of traumatic stress.

### 3.2 Effect of trauma on perinatal outcomes

Prior traumatic experiences can have lasting effects on mental and physical perinatal outcomes. Patients with a history of childhood trauma (e.g., emotional or physical abuse, loss of a parent) or current life stressors (e.g., financial strain, loss of employment) are at a significantly increased risk of IPV during pregnancy (adjusted odds ratio [aOR] = 2.48; 95% CI: 1.46–4.22), and the risk increases with three or greater adverse childhood experiences (ACEs) (aOR = 4.40; 95% CI: 2.52–7.67) [56, 57]. Additionally, these individuals are more likely to have higher levels of mistrust with providers and inadequate utilization of prenatal care, limiting their opportunities for support and resources [58, 59, 60]. During labor and delivery, patients who have experiences TLEs have an increased risk of obstetric violence and childbirth‐associated PTSD highlighting a particular vulnerability to re‐traumatization [22, 52, 61, 62, 63, 64]. In the postpartum period, prior traumatic experiences have been shown to be a strong predictor of postpartum mood disorders [57, 65, 66, 67, 68, 69]. In one survey of 46 patients, postpartum individuals who were hospitalized for severe perinatal mental health disorders, 76% had some form of lifetime trauma exposure and 24% qualified for a diagnosis of complex PTSD, rates much higher than the general population (22.7% and 0.5% respectively) [70].

Besides negative mental health outcomes, traumatic stress has been associated with multiple adverse health outcomes during the perinatal period. Patients with a history of multiple ACEs have been shown to have an increased risk of pregnancy loss (relative risk [RR] = 1.96; 95% CI: 1.20–3.20) and infertility (RR = 2.75; 95% CI: 1.45–5.21) [71]. Additionally, childhood maltreatment has been associated with increased rates of complications during pregnancy, such as decreased sleep quality, substance use, hypertensive disorders, and gestational diabetes (Table 2) [57, 72, 73, 74, 75, 76, 77]. Traumatic experiences prior to pregnancy and stressors during pregnancy can also increase various neonatal risks including pre‐term birth and low birth weight (Table 2) [75, 78, 79, 80].

**TABLE 2:** Effect of prior traumatic life events in the perinatal period. This table illustrates the physical perinatal risks that have been associated with a history of trauma prior to pregnancy.

Outcome	Effect size [95% confidence interval]
Poor sleep quality	aOR = 2.11 [1.35–3.30] [73]
Any substance use during pregnancy	aOR = 1.72 [1.12–2.65] [57]
Cigarette smoking during pregnancy	aOR = 17.57 [1.59–194.6] [72]
Gestational diabetes	RR = 1.37 [1.02–1.83] [74] OR = 1.39 [1.11– 1.74] [75]
Hypertensive disorders of pregnancy	aOR = 1.55 [1.06–2.26] [77]
Low neonatal birth weight	OR = 1.42 [1.10–1.83] [74] OR 1.27 [1.02–1.47] [75]
Pre‐term delivery	OR = 1.27 [1.06–1.52] [74] OR = 1.41 [1.16–1.71] [75]

A history of trauma, particularly emotional abuse in childhood and childbirth‐associated trauma, can also lead to issues with attachment and interpersonal relationships, often creating a generational cycle of traumatic life experiences [63]. Studies have shown that childhood abuse and trauma is linked to struggling with the adjustment to motherhood, particularly due to a lack of confidence in their own parenting skills and difficulties with mother‐infant bonding [81]. One qualitative study found that women with higher levels of traumatic stress and lower support often faced greater challenges with breastfeeding, with many unable to continue for as long as they had hoped [82]. These factors all play into maternal‐child attachment, which has long lasting implications for childhood development. Children of mothers with PTSD demonstrated significantly higher scores on the Ages and Stages Questionnaire: Social‐Emotional (ASQ:SE), with a mean increase of 3.6 points (95% CI: 1.8, 5.4), indicating greater social‐emotional difficulties [83]. These children have also exhibited increased symptoms of ADHD (β = 0.33, *p* = 0.014), PTSD (β = 0.48, *p* < 0.001), and other psychological disorders [84, 85].

### 3.3 Implementation of trauma‐informed care

TIC is widely endorsed as a best practice, yet its integration into OB/GYN training remains inconsistent, and implementation varies significantly across settings. At the core of TIC are the “Four R's”: **R** ealizing the prevalence and impact of trauma, **R** ecognizing its signs, **R** esponding with evidence‐based interventions, and **R** esisting re‐traumatization by centering patient autonomy and care preferences [2]. We propose an addition to this model—fostering patient **R** esilience (Table 3). Having examined the prevalence and pervasive effects of trauma on perinatal health and outcomes, it is important to focus on specific strategies to enhance its implementation in clinical practice.

**TABLE 3:** The “Four R's” of trauma‐informed care + Resilience. This table outlines key actions and examples associated with the “Four R's” of trauma‐informed care—Realize, Recognize, Respond, and Resist re‐traumatization—with the addition of a fifth element, Resilience. Adapted from SAMHSA's *Concept of Trauma and Guidance for a Trauma‐informed Approach* [2].

	Action	Example
**Realize**	**Train healthcare providers** on the prevalence and impact of trauma on maternal and neonatal health, including how trauma may affect emotional responses and care needs.	Residents and medical students receive training on trauma prevalence and its impact on perinatal health. The training emphasizes how trauma shapes patient responses and care preferences.
**Recognize**	**Screen for traumatic stress** and recognize signs of distress, ensuring care approaches are adjusted accordingly.	The provider notices a patient withdrawing during a pelvic exam and gently asks about how she is feeling, prioritizing her comfort and autonomy during the procedure.
**Respond**	**Respond with empathy and calmness**, validating the patient's experience and acknowledging their trauma history. Interpret “difficult” behaviors in the context of prior adverse experiences.	A patient hesitates to consent to Pitocin, citing a traumatic prior delivery. The provider listens empathetically, explains the benefits and risks, and ensures the patient feels empowered to make an informed choice.
**Resist re‐traumatization**	**Avoid re‐traumatizing practices**, prioritize patient autonomy, and use language that is sensitive and reassures patients.	During a cervical exam, the provider describes each step beforehand, checks in frequently, and respects the patient's ability to stop the procedure.
**Resilience**	**Build patient resilience** by identifying strengths, encouraging support networks, and offering resources for ongoing emotional support and coping.	A patient with sexual trauma receives a referral to a trauma‐informed counselor and collaborates on a personalized birth plan to address triggers and incorporate support during labor.

Recognizing the symptoms of traumatic stress is fundamental to implementing TIC, as it requires providers to adopt a trauma‐informed lens—moving beyond surface‐level interpretations of patient behaviors to understanding them within the context of past trauma and potential triggers. For example, a patient who initiates prenatal care late in pregnancy may not be negligent or nonadherent, but instead face barriers such as lack of transportation, limited health literacy, or avoidance of medical settings due to past trauma, such as experiences of discrimination in healthcare (Table 4). Recognizing and acknowledging the reasons for a patient's perceived “difficult” behavior can help patients feel more understood and at ease [86]. Education about this contextual lens has been shown to enhance provider understanding and reduce bias in interpreting patient responses [87, 88]. TIC also calls for providers to reflect on how their own behaviors, language, or clinical practices may inadvertently trigger distress or re‐traumatization in patients. Something as routine as a pelvic exam, use of medical jargon, or a rushed interaction may evoke feelings of powerlessness or fear in someone with a trauma history. Developing this self‐awareness is essential to fostering a sense of safety and trust in the clinical encounter.

**TABLE 4:** Examples of traumatic stress symptoms in perinatal care. This table presents illustrative examples of how traumatic stress symptoms may manifest in the perinatal setting. Adapted from DSM‐5 criteria for the diagnosis of PTSD [89].

Symptom	Example
**Intrusive thoughts/flashbacks**	During a non‐stress test, the patient hears the beeping of the monitor, which triggers vivid memories of the constant alarms during a previous traumatic delivery. She becomes visibly tense and stops responding to questions.
**Hypervigilance**	A patient with a history of intrauterine fetal demise uses a home doppler multiple times a day to monitor her current baby's heartbeat, unable to find reassurance without constant checks.
**Avoidance**	Due to experiencing coercive and disrespectful care in her previous childbirth, a patient delays beginning prenatal care, attempting to avoid triggering interactions.
**Negative emotions**	When asked about a history of sexually transmitted infections (STI), the patient becomes visibly upset and raises her voice, saying, “That's none of your business!” She is reluctant to reveal that she contracted an STI during a sexual assault years ago.
**Dissociation**	During a pelvic examination, a patient with a history of childhood sexual abuse disengages entirely. Her gaze fixes on the ceiling, and she remains unresponsive to the provider's attempts to communicate.

**FIGURE 1:**
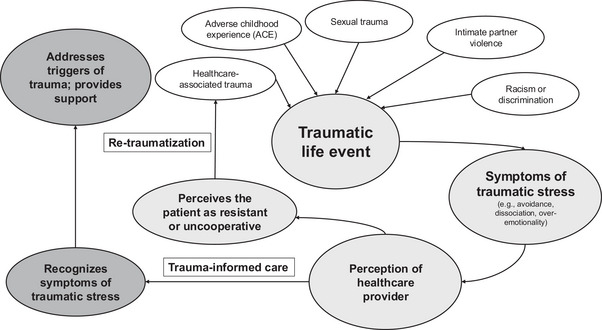
Trauma‐informed care model for preventing re‐traumatization. A flow diagram illustrating how traumatic life events manifest as trauma symptoms, with provider stigma perpetuating re‐traumatization. Trauma‐informed care disrupts this cycle by reducing bias and enhancing recognition and response to traumatic stress.

Response to patients’ expressions of traumatic stress is crucial for fostering the patient‐provider relationship and avoiding re‐traumatization. Pregnant individuals often describe low‐quality provider interactions as impersonal, objectifying, and disempowering, particularly when they feel a lack of control over their bodies [51, 90, 91]. While labor is inherently unpredictable, and many interventions are invasive by necessity, evidence suggests that the way care is delivered—rather than the interventions themselves—has a greater impact on psychological outcomes for trauma survivors. Negative interactions, such as withholding or manipulating information, disregarding patient requests, and repeatedly ignoring concerns about pain management, can contribute to distress and feelings of helplessness, and these interactions more prevalent for patients of color [92, 93, 94]. Importantly, these behaviors are rarely the result of malice or ill intent on behalf of the provider but instead reflect stress, time constraints, or systemic pressures. However, small but intentional actions—such as pausing to acknowledge a patient's concerns, providing clear explanations of procedures, and actively involving them in decision‐making—can be profoundly healing and empowering for survivors of trauma, and avoid the cycle of re‐traumatization (Figure 1).

Resilience has been shown to buffer the effects of traumatic life experiences and improve interpersonal and psychological outcomes [95, 96, 97]. High levels of resilience are likely to reduce perinatal stress, anxiety, and depression, as well as disrupt the intergenerational cycle of trauma for their infants [57, 98, 99]. Resilience even buffers the negative mental health effects of additional stressors, such as food insecurity [100]. A key contributor to resilience, particularly among pregnant individuals with a history of trauma, is the presence of strong support networks. Indeed, supportive relationships during childhood, whether with parents, caregivers, or other trusted adults—can play a critical role in shaping this resilience and buffering the effects of trauma [101]. Strategies to foster such support in perinatal care include actively involving trusted family members and supporting people in visits and care plans [86]. Connecting patients with peer support and group prenatal care has also demonstrated meaningful benefits, including emotional validation and the exchange of coping strategies [102, 103]. Group prenatal care helps high risk patients reduce their distress surrounding pregnancy, increase coping strategies, and improve their maternal functioning in the postpartum period [104, 105]. In addition, the presence of doulas has been shown to buffer the effects of trauma and negative birth experiences, particularly those from racially or culturally marginalized backgrounds [106]. Beyond emotional support, doula care has been shown to lower the risk of cesarean delivery by 47% (RR  =  0.53; 95% CI  =  0.43, 0.66) and the risk of preterm birth by 29% (RR  =  0.71; 95% CI  =  0.51, 0.98) [107].

Previously implemented TIC strategies and initiatives have shown success when it comes to improving patient outcomes and resilience. Many groups are shifting towards a frame of “healing‐centered engagement,” which utilizes the tenants of TIC in a more strengths‐based and holistic approach. This model promotes comprehensive mental health support, physical wellness, and peer connection, and have been associated with increased engagement in prenatal care and reduced risk of adverse outcomes such as low birth weight [108, 109]. Several clinics and community initiatives have worked to provide multidisciplinary care, with obstetrics, psychiatry, and social work all working in tandem to provide care for the pregnant person and their fetus. Building a community of care providers and fellow patients offers multiple sources of support to pregnant individuals, providing social connections and making care more accessible.

Although there is significant evidence to support the implementation of TIC, and providers have a positive view of such strategies, there remain significant barriers to implementation. Lack of time, inadequate referral options, and lack of training are commonly reported factors that prevent clinicians from employing TIC principles [110, 111]. Studies have taken strides to address these concerns, with one utilizing pre‐clinic screening checklists. This initiative improved the disclosure of trauma and detection of PTSD, better allowing providers to identify patients who need extra resources [112]. However, universal screening is not without risk. It can be re‐traumatizing for some patients, particularly if questions are not posed sensitively or if appropriate support is not readily available. As such, screening for trauma should only be undertaken when adequate resources are in place to respond to identified needs. To help address these implementation challenges and promote confidence in delivering TIC, multiple educational approaches have been effective in increasing faculty and trainee confidence in applying TIC. These include simulations, role‐play with communication feedback, and brief seminars covering core TIC principles [113, 114, 115].

Using the principles and evidence discussed, TIC practices can be implemented individually and institutionally (Tables 5 and 6). Using validated screening tools as pre‐visit checklists can assess patient trauma histories and provide equitable, time‐efficient care. Physicians can advocate for TIC education at their institutions, offering programs for trainees and continuing education for staff on the identification of traumatic stress. It is important to ensure that education is put into practice by providing example scripts for communication and role‐playing scenarios to practice emotional responses. Clinicians can individualize this care for their community and patients, such as maintaining a list of support groups, identifying effective referral options, and recognizing patient resilience factors. Finally, seek continuous feedback from trauma survivors to refine and improve these care strategies.

**TABLE 5:** Example strategies and language for trauma‐informed pelvic examinations. This table provides brief practice points and suggested language to support trauma‐informed approaches for conducting pelvic examinations.

**Prioritize patient autonomy and offer alternatives or accommodations when feasible**	‐ Emphasize that the patient maintains control and can pause or stop the exam at any point. ‐ Provide alternatives to using the footrests, such as allowing the patient to place their feet on the edge of the examination table or place their feet together [24]. ‐ Offer self‐insertion of the speculum, ultrasound probe, etc.
**Before starting any part of the exam, explain your actions using neutral, non‐threatening language, provide a clear rationale for each step, and set expectations for any sensations the patient may experience**.	‐ “First, I will examine the external genitalia to check for infections or lesions, and you will feel my hands on the outside of your vulva.” ‐ “We will be inserting a speculum to visualize the cervix, so you feel pressure in your vagina.” ‐ “Now I will insert two fingers into your vagina to feel the position of your uterus and check for pelvic masses. You will feel some pressure.”
**Avoid triggering language**	‐ “Open your knees for me.” ‐ “Relaxing will make it hurt less.” [116]

**TABLE 6:** Barriers to trauma‐informed care and proposed implementation strategies. This table outlines common provider‐identified barriers to implementing trauma‐informed care, along with potential strategies to address them.

Barrier	Proposed solution
**Lack of time**	Use pre‐visit trauma screening checklists to streamline identification. Incorporate TIC education into existing meetings (e.g., morning huddles).
**Lack of confidence or experience**	Implement TIC training through role‐play, simulation, or brief online modules. Provide scripted language examples for trauma‐sensitive communication.
**Inadequate or too few referral options**	Develop a tiered referral system (e.g., in‐house mental health support, community partnerships, local support groups). Train providers in brief crisis interventions when referrals are unavailable.

### 3.4 Professional society guidelines and available curricula

Major professional organizations provide recommendations and guidance on the implementation of TIC strategies.

The Substance Abuse and Mental Health Services Administration (SAMHSA) updated the “Practical Guide for Implementing a Trauma‐Informed Approach” in 2023 [117]. This is a free, online publication that seeks to “[recognize] that substance use disorders and mental illnesses are often rooted in structural inequities and influenced by the social determinants of health.” Its comprehensive approach emphasizes leadership engagement, policy development, and workforce support to implement trauma‐informed systems of care.

The American College of Obstetricians and Gynecologists (ACOG) incorporates a similar stance in their committee opinion “Caring for Patients Who Have Experienced Trauma” [118]. ACOG provides specific patient‐provider interaction strategies, including asking for permission before exams, describing procedures in advance, and minimizing re‐traumatization through gentle and respectful clinical practices that centralize autonomy. It also advocates for universal trauma screening and structured training about trauma for obstetric clinicians and trainees.

The Society for Maternal‐Fetal Medicine (SMFM) focuses its TIC guidance on confronting structural racism and inequities in maternal health [119, 120, 121]. SMFM distinguishes itself by framing the experience of trauma through a health equity lens, emphasizing the need for culturally responsive care and systemic change to reduce maternal morbidity and mortality—particularly for historically marginalized populations.

Lamaze International has several modules for TIC that are available to perinatal providers [122]. Their unique curriculum centers on childbirth education and the patient experience, with content addressing perinatal mood and anxiety disorders, obstetric violence, and racial disparities in maternal outcomes. Lamaze's approach supports providers in creating emotionally safe, empowering birthing environments.

Postpartum Support International also provides webinars and curricula to help providers improve their ability to address postpartum mental health concerns, substance use, and provide culturally competent care [123].

## 4 CONCLUSIONS

Trauma and traumatic stress are highly prevalent in the obstetric population, with disproportionate impacts on marginalized communities who face higher rates of violence, structural inequities, and discrimination. Unaddressed traumatic stress can contribute to worsened maternal and fetal outcomes, including elevated risks for mental health complications and obstetric morbidity. As healthcare providers, we have both an opportunity and a responsibility to implement TIC practices—at the individual and institutional levels—to improve patient‐centered care, support resilience, and promote healing. Recognizing and responding to trauma is not only essential for clinical excellence but also for advancing health equity and reducing the disparities that drive maternal morbidity and mortality in vulnerable populations. TIC is a foundational approach to equitable, respectful, and effective perinatal health delivery.

## CONFLICT OF INTEREST STATEMENT

The authors declare no conflicts of interest.

## Supporting information



Supporting Information
