# Supplementary material for: Reframing obstetric care through a trauma‐informed lens: A narrative review of trauma‐informed principles and clinical applications
Source: Pregnancy (Hoboken). 2025 Aug 5;1(5):e70081. doi: 10.1002/pmf2.70081 (PMC13344757; doi:10.1002/pmf2.70081)
Supplement: Supplementary file 1 [file PMF2-1-e70081-s001.docx]

**Supplement 1. SANRA Quality Assessment of the Narrative Review.** The Scale for the Assessment of Narrative Review Articles (SANRA) was used to evaluate key aspects of the review, including justification of importance, clarity of aims, literature search description, referencing, scientific reasoning, and data presentation.^28^ Each item was scored from 0 (low quality) to 2 (high quality), with the overall score indicating a high-quality narrative review (11.5/12). The “Presentation of Data” score was somewhat lower due to a reliance on narrative synthesis over detailed quantitative data.

| **SANRA Item** | **Description** | **Score (0-2)** |
| --- | --- | --- |
| **Justification of the article’s importance** | Clear rationale for the review topic and its relevance. | 2 |
| **Statement of concrete aims or formulation of questions** | Clear aims and research questions. | 2 |
| **Description of the literature search** | Transparent and reproducible search strategy. | 2 |
| **Referencing** | Appropriate and comprehensive referencing throughout. | 2 |
| **Scientific reasoning** | Logical argumentation, synthesis of evidence. | 2 |
| **Presentation of relevant and appropriate data** | Inclusion of key data, tables, and critical analysis. | 1.5 |
